# Connectome sorting by consensus clustering increases separability in group neuroimaging studies

**DOI:** 10.1162/netn_a_00074

**Published:** 2019-02-01

**Authors:** Javier Rasero, Ibai Diez, Jesus M. Cortes, Daniele Marinazzo, Sebastiano Stramaglia

**Affiliations:** Biocruces Health Research Institute, Hospital Universitario de Cruces, Barakaldo, Spain; Functional Neurology Research Group, Department of Neurology, Massachusetts General Hospital, Harvard Medical School, Boston, MA, USA; Gordon Center, Department of Nuclear Medicine, Massachusetts General Hospital, Harvard Medical School, Boston, MA, USA; Neurotechnology Laboratory, Tecnalia Health Department, Derio, Spain; Biocruces Health Research Institute, Hospital Universitario de Cruces, Barakaldo, Spain; Department of Cell Biology and Histology, University of the Basque Country, Leioa, Spain; Ikerbasque, The Basque Foundation for Science, Bilbao, Spain; Faculty of Psychology and Educational Sciences, Department of Data Analysis, Ghent University, Ghent, Belgium; Dipartimento di Fisica, Universitá degli Studi “Aldo Moro” Bari, Italy; Istituto Nazionale di Fisica Nucleare, Sezione di Bari, Italy; TIRES-Center of Innovative Technologies for Signal Detection and Processing, Universitá degli Studi “Aldo Moro” Bari, Italy

**Keywords:** Unsupervised learning, Consensus clustering, Brain connectivity, Classification

## Abstract

A fundamental challenge in preprocessing pipelines for neuroimaging datasets is to increase the signal-to-noise ratio for subsequent analyses. In the same line, we suggest here that the application of the consensus clustering approach to brain connectivity matrices can be a valid additional step for *connectome processing* to find subgroups of subjects with reduced intragroup variability and therefore increasing the separability of the distinct subgroups when connectomes are used as a biomarker. Moreover, by partitioning the data with consensus clustering before any group comparison (for instance, between a healthy population vs. a pathological one), we demonstrate that unique regions within each cluster arise and bring new information that could be relevant from a clinical point of view.

## INTRODUCTION

In the supervised classification of human connectome data (Sporns, 2011), subjects are usually grouped based on high-level clinical categories (e.g., patients and controls, early vs. late cognitive impairment, etc.), and typical approaches aim at deducing a decision function from the labeled training data (see, e.g., Fornito & Bullmore, 2010). Likewise, unsupervised analysis is also usually performed with the researcher being blind to any phenotypic factors and aiming to find subgroups of subjects/features with similar characteristics. The existing literature reports several clustering algorithms dealing with these issues (see, e.g., Xu & Wunsch, 2005, and references within). The emergence of substructures underlying the data is very often due to the fact that the populations to be studied (healthy subjects, patients, etc.) are usually highly heterogeneous. Consequently, stratification of groups may be a useful preliminary step, allowing the subsequent supervised analysis to exploit the knowledge of the data’s structure.

A convenient strategy for group stratification involves using subjects’ phenotypic variables when available (for instance, the presence or absence of one specific gene mutation). More interestingly, stratification may also rely on the measured variables, like the human connectome data itself, applying clustering algorithms to find natural groupings in the data.

An effective supervised approach, named multivariate distance matrix regression (MDMR), has been proposed in Zapala & Schork (2006) for the analysis of gene expression patterns; it tests the relationship between variation in a distance matrix and predictor information collected on the samples whose gene expression levels have been used to construct the matrix. The same method was also applied to the cross-group analysis of brain connectivity matrices (Shehzad et al., 2014), as an alternative to the very commonly used method of connectome-wide association, that is, mass-univariate statistical analyses, in which the association with a phenotypic variable of each entry of the brain connectivity matrix across subjects is tested. While MDMR has found wide application (see, for example, Rasero et al., 2017), these findings may be certainly affected by the heterogeneity of classes.

Recently an unsupervised method (Rasero et al., 2017), rooted on the notion of *consensus* clustering (Lancichinetti & Fortunato, 2012), has been developed for community detection in complex networks (Barabasi & Frangos, 2002), when a connectivity matrix is associated with each item to be classified. In this method, the different features, extracted from connectivity matrices, are not combined into a single vector to feed the clustering algorithm; rather, the information coming from the various features is combined by constructing a *consensus* network (Lancichinetti & Fortunato, 2012). Consensus clustering is commonly used to generate stable results out of a set of partitions delivered by different clustering algorithms (and/or parameters) applied to the same data (Strehl & Ghosh, 2002). In the method developed in Rasero et al. (2017), instead the consensus strategy was used to combine the information about the data structure arising from different features so as to summarize them in a single consensus matrix. Furthermore, such a matrix not only provides a partition of subjects in communities, but also a geometrical representation of the set of subjects. It has been shown that this technique can be effective in disentangling the heterogeneity of a population. The main similarity with MDMR is that in both methods a distance matrix in the space of subjects is introduced.

The purpose of this work is to propose the consensus clustering approach from Rasero et al. (2017) as a step to perform prior to MDMR in exploratory analysis, so as to cope with the heterogeneity present in user-defined groups. First, we will test the robustness of our consensus clustering by using noise to introduce heterogeneity in the data. Second, we will show that extracting the natural classes present in the user-defined groups and subsequently performing the supervised analysis between the subgroups found by consensus clustering allows us to identify variables whose patterns are altered in group comparisons, which are not identified when the groups are used as a whole. As a result, the proposed approach leads to an increase in separability.

## MATERIALS AND METHODS

### Materials

The robustness of the consensus clustering algorithm to disentangle the real structure of data was tested using simulated data for two groups of 15 subjects each, generated using *simTB* (Erhardt, Allen, Wei, Eichele, & Calhoun, 2012), a toolbox written in MATLAB that allows simulation of functional magnetic resonance imaging (fMRI) datasets under a model of spatiotemporal separability. Given the number of sources/components (*n*_*C*_), repetition time (TR), and a hemodynamic model, the program yields the time course profile subject to the experimental design (block- and/or event-related) provided. Our scenario consists of *n*_*C*_ = 20 components and *TR* = 2 s, such that in an event-related experiment the first 10 components of the cohort of group 1 have high probability (90%) of becoming activated, whereas for the remaining components the activation is rare (10%). For subjects of group 2 the situation is the opposite. This scenario could be then considered as the same group of subjects performing two orthogonal tasks, where different components get involved. The length of the simulated time series is 148 points.

The advantages of using the consensus clustering method as a sorting preliminary step were explored on two real and public neuroimaging datasets. The first dataset comprises healthy controls (HC) and individuals diagnosed with three pathologies: attention-deficit hyperactivity disorder (ADHD), bipolar disorder (BD), and schizophrenia (SCH). This dataset is provided by the Consortium for Neuropsychiatric Phenomics LA5C Study and was downloaded from the OpenfMRI database with accession number ds000030 (Poldrack et al., 2016). We used resting functional MRI (rfMRI) of 255 subjects: 40 ADHD, 47 BD, 50 SCH, and 118 HC. Demographics information about this cohort can be found in Table 1. Data were preprocessed with FSL (FMRIB Software Library v5.0). All volume images were corrected for motion, after which slice timing correction was applied to correct for temporal alignment. All voxels were spatially smoothed with a 6-mm full width at half-maximum isotropic Gaussian kernel, and a band-pass filter was applied between 0.01 and 0.08 Hz after intensity normalization. In addition, linear and quadratic trends were removed. We next regressed out the motion time courses, the average CSF signal and the average white matter signal. Global signal regression was not performed. Data were transformed to the MNI152 template, such that a given voxel had a volume of 3 mm × 3 mm × 3 mm. Finally, we obtained 278 time series, each corresponding to an anatomical region of interest by averaging the voxel signals according to the functional atlas described in (Shen, Tokoglu, Papademetris, & Constable, 2013). Finally, a 278 × 278 matrix of Pearson coefficients among time series for each subject was obtained.

**Table 1.** Demographic information for LA5C dataset

	Sex (M/F)	Age	FWD
HC (*N* = 118)	64/54	31.56 ± 8.83	0.17 ± 0.15
ADHD (*N* = 40)	21/19	32.05 ± 10.4	0.15 ± 0.11
BD (*N* = 47)	26/21	35.47 ± 9.16	0.19 ± 0.13
SCH (*N* = 50)	38/12	36.46 ± 8.88	0.25 ± 0.20

*Note*. FWD = framewise displacement; HC = healthy controls; ADHD = attention-deficit hyperactivity disorder; BD = bipolar disorder; SCH = schizophrenia.

As for the second dataset, it contains resting-state fMRI scans from a population of autism spectrum disorder (ASD) subjects and a population of typically developing (TD) ones, from the Autism Brain Imaging Data Exchange (ABIDE) repository (Di Martino et al., 2014), an initiative that aims at collecting data from laboratories around the world to accelerate the understanding of the neural bases of this disorder. Since it is unclear what effect that different scanner machines can have on the observable results, we decided to avoid this additional possible confounder by considering only a sample of 75 subjects for each group acquired at the same site (NYU). For this subset, we matched age and sex between samples (Wilcoxon rank sum *p* = 0.27 and *χ*^2^ test *p* = 0.29, respectively). Preprocessing of the data was done using FSL, AFNI, and MATLAB. First, slice-time correction was applied. Then, each volume was aligned to the middle volume to correct for head motion artifacts followed by intensity normalization. We next regressed out 24 motion parameters, the average cerebrospinal fluid (CSF) and the average white matter signal. A band-pass filter was applied between 0.01 and 0.08 Hz, and linear and quadratic trends were removed. All voxels were spatially smoothed with a 6-mm FWHM. Finally, FreeSurfer software was used for brain segmentation and cortical parcellation. An 86 region atlas was generated with 68 cortical regions from the Desikan-Killiany Atlas (34 in each hemisphere) and 18 subcortical regions (9 in each hemisphere: thalamus, caudate, putamen, pallidum, hippocampus, amygdala, accumbens, ventralDC and cerebellum). Each subject parcellation was projected to individual functional data, and the mean functional time series of each region was computed.

### Consensus clustering method

Given a set of matrices of distance among subjects, the consensus clustering proposed in (Rasero et al., 2017) can be summarized as follows: (i) cluster each distance matrix using a known clustering algorithm, (ii) build the consensus network from the corresponding partitions and (iii) extract groups of subjects by finding the communities of the consensus network thus obtained.

Regarding the distance matrices calculation in the case of fMRI data, let us consider *m* subjects such that each one has a *N* × *N* matrix of features, which can for example represent the brain functional connectivity matrix. We will denote this matrix as {**A**(*i*, *j*)_*α*_}, where *α* = 1, …, *m* and *i*, *j* = 1, …, *N*. For each row *i*, we build a distance matrix for the set of subjects as follows. Consider a pair of subjects *α* and *β*, and consider the corresponding patterns {**A**(*i*, :)_*α*_} and {**A**(*i*, :)_*β*_}; let *r* be their Pearson correlation. As the distance between the two subjects, for the node *i* we take *d*_*αβ*_ = 1 − *r*; other choices for the distance can be used, like, for example, $d_{\alpha \beta }=\sqrt{2(1-r)}$, where *r* is the Pearson correlation. The *m* × *m* distance matrix *d*_*αβ*_ corresponding to row *i* will be denoted by **D**_**i**_, with *i* = 1, …, *N*. The set of **D** matrices may be seen as corresponding to layers of a multilayer network (Boccaletti et al., 2014), each brain node representing a layer.

Each distance matrix **D**_**i**_ is then partitioned into *k* groups of subjects using the *k*-medoids method (Brito, Bertrand, Cucumel, & Carvalho, 2007), and this cluster information is encoded into an adjacency matrix among subjects. Subsequently, an *m* × *m* consensus matrix **C** is evaluated by averaging this different information across the nodes. Hence, the entries of *C*_*αβ*_ indicate the number of partitions in which subjects *α* and *β* are assigned to the same group, divided by the number of partitions *N*. Eventually, the consensus matrix is averaged over *k* ranging in the interval (2–20) so as to combine, in the final consensus matrix, information about structures at different resolutions (for more details, see Rasero et al., 2017).

The consensus matrix obtained as explained before provides a weighted graph that contains all the community information, whose structure can be further examined by an appropriate method. Following Rasero et al. (2017), we perform this by modularity optimization, for which we compared this consensus matrix with the ensemble of all consensus matrices that one may obtain randomly and independently by permuting the cluster labels obtained after applying the *k*-medoids algorithm to each of the set of distance matrices. More precisely, a modularity matrix is evaluated as

B=C−γ⋅P, (1)

where **P** is the expected co-assignment matrix, uniform as a consequence of the null ensemble here chosen, obtained repeating many times the permutation of labels, and *γ* is the resolution parameter that all modularity matrices implicitly or explicitly carry. As in the Newman and Girvan scenario, we hereafter set this parameter to be 1, which corresponds to the maximum modularity case (Reichardt & Bornholdt, 2006).

A modularity matrix of this kind encodes all the information at different levels about the interaction strength among subjects. As a result, one could now just as easily define a distance matrix based on this matrix by computing any metric distance between pairs of rows and follow with *k*-medoids to perform a clustering analysis. We instead submitted this matrix to a modularity optimization algorithm, which does not require a predefined number of clusters, to obtain the output partition by the proposed approach (we used the Community Louvain routine in the Brain Connectivity Toolbox (Rubinov & Sporns, 2010), which admits modularity matrices instead of connectivity matrices as input.

### Multivariate Distance Matrix Regression

The cross-group analysis of brain connectivity matrices has been performed using the MDMR approach as described in Shehzad et al. (2014) and Zapala & Schork (2006). This method allows for an identification of voxels/regions whose whole-brain connectivity patterns vary significantly with a given set of regressor variables. In contrast to the usual general linear regression model, the variation on the total sum of squares here is tested on the Distance matrix, constructed from the connectivity patterns for each node, rather than directly on the observed data. This approach can assess significance of multiple connections at the same time and is especially useful when the number of dependent variables—in this case, the entries of the connectivity matrices—exceeds the number of observations.

For a fixed brain node *i*, the distance between connectivity patterns of *i* with the rest of the brain was calculated per pair of subjects (*u*, *v*), by calculating Pearson correlation between connectivity vectors of subject pairs, thus leading to a distance matrix in the subject space for each *i* investigated. In particular, the following formula was applied

$$d_{uv}^{i}=\sqrt{2\left(1-r_{uv}^{i}\right)}$$ (2)

where $r_{uv}^{i}$ is the Pearson correlation between connectivity patterns of node *i* for subjects *u* and *v*. Next, MDMR was applied to perform cross-group analysis as implemented in R (McArtor, n.d.).

MDMR yielded a pseudo-*F* estimator (analogous to that *F* estimator in standard ANOVA analysis), which addresses significance due to between-group variation as compared with within-group variations (McArdle & Anderson, 2001) on a distance matrix calculated from a set of variables. In the particular case when the only regressor variable is categorical (i.e., the group label), given a distance matrix for a certain node, one can calculate the total sum of squares as

$$SS_{T}=\frac{1}{n}\overset{N}{\underset{u=1}{\sum }}\overset{N}{\underset{v=u+1}{\sum }}d_{uv}^{2}$$ (3)

with *N* being the total number of subjects. Similarly, the within-group sum of squares can be written as

$$SS_{W}=\sum \frac{1}{n_{g}}\underset{u=1}{\sum }\overset{n}{\underset{v=u+1}{\sum }}\overset{2}{\underset{uv}{d}}\overset{g}{\underset{uv}{\epsilon }}$$ (4)

where *n*_*g*_ is the number of subjects per group and $\epsilon_{uv}^{g}$ a variable equal to 1 if subjects *u* and *v* belong to group *g* and 0 otherwise. The between-group variation is simply *SSB* = *SST* − *SSW*, which leads to a pseudo-*F* statistic as follows

$$F=(N-1)\frac{SS_{A}}{SS_{W}},$$ (5)

where *m* is the number of groups. As it was acknowledged in Zapala & Schork (2006), the pseudo-*F* statistic is not distributed like the usual Fisher’s *F*-distribution under the null hypothesis. Accordingly, we randomly shuffled the subject indexes and computed the pseudo-*F* statistic each time. A *p* value is computed by counting those pseudo-*F* statistic values from permuted data greater than that from the original data respect to the total number of performed permutations.

Nevertheless, in our cross-group analyses, in addition to group label, age, sex, and mean framewise displacement (FWR) were also considered as covariates because of their possible confounding effect in distance variation between subjects. Finally, we controlled for I errors by false discovery rate corrections and set significance threshold at 1%.

### The proposed approach

The application of MDMR to identify altered patterns is hampered by the heterogeneity of subjects in the same group (healthy controls or patients). Therefore, we propose here the use of the consensus clustering approach as a sorting preliminary tool. In a first step, the consensus clustering is to be applied to extract the natural classes present in the group of controls (and/or in the group of patients). In a subsequent step, the supervised analysis of MDMR is to be performed between the pairs of subgroups found by consensus clustering. In this way one can identify variables whose pattern is altered in subgroups comparisons, but not in the whole group.

## RESULTS

### Simulated data

**Figure 1.**
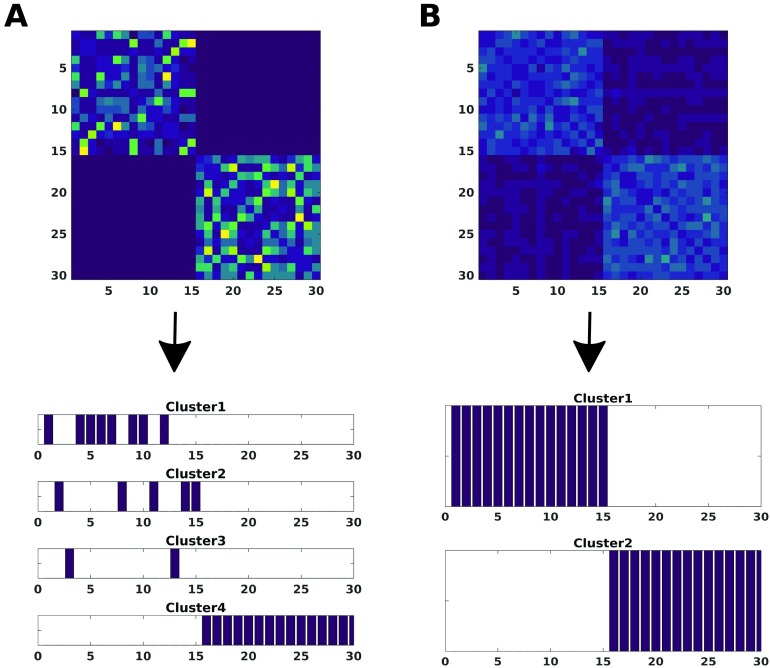
The modularity matrix among subjects of both groups and its cluster structure reconstruction provided by the Louvain community detection algorithm on this matrix is depicted in two different scenarios: Application of the consensus clustering to the distance matrix from the whole pattern connectivity matrix (A) or to the set of distance matrices from each node pattern connectivity (B).

Our approach aims to use cluster information from each node instead of using the whole set of nodes across the brain. We can visualize this in Figure 1, where the group reconstruction provided by our method is compared with the one yielded by considering the whole correlation matrix as pattern connectivity from which one calculates the distance matrix, so the average is only carried out over different resolutions *κ* when applying *k*-medoids. Therefore, the introduction of a clustering step at a node-level resolution seems to permit a better cluster reconstruction, confirming the findings in Rasero et al. (2017).

We also assessed the robustness of our method in group reconstruction when Gaussian noise is added to the time series of group 1 (*TS*_1_) and group 2 (*TS*_2_) as follows:

TS1ij=TS1ij+ϵ1ij⋅A⋅N1ij(0,1)TS2ij=TS2ij+ϵ2ij⋅A⋅N2ij(0,1), (6)

for *i* = 1…15 subjects and *j* = 1…20 components. *A* is the amplitude of the perturbations and $\epsilon_{1,2}^{ij}$ is a binary matrix allowing us to play with different noise configuration scenarios. In our notation, subscript index represents group label and superscript indices *i*, *j* subject and component, respectively.

**Figure 2.**
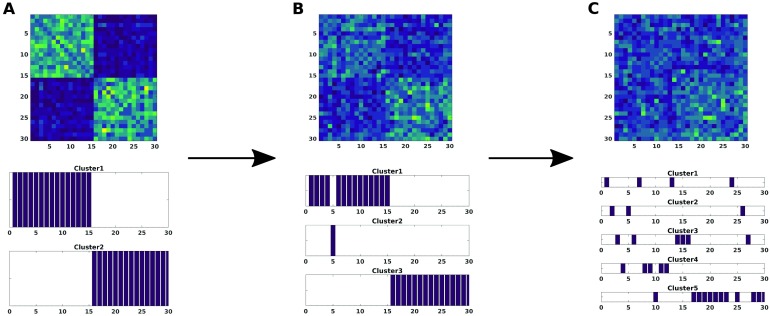
The modularity matrix among subjects of both groups and its cluster structure reconstruction provided by a Louvain community detection algorithm on this matrix is depicted when changing the amplitude noise *A* in Equation 6, that affects all the simulated time series for both groups: *A* = 0.1 (A), *A* = 0.3 (B), *A* = 0.5 (C).

In a first scenario, different values of the noise amplitude *A* = {0.1, 0.3, 0.5} were applied to all components and subjects $\epsilon_{1,2}^{i=1..15,j=1..20}=1$. As we can see in Figure 2, only for large amplitude, noise starts to dominate so much that the consensus algorithm renders group more compact and mix them together making it incapable of distinguishing between groups perfectly.

**Figure 3.**
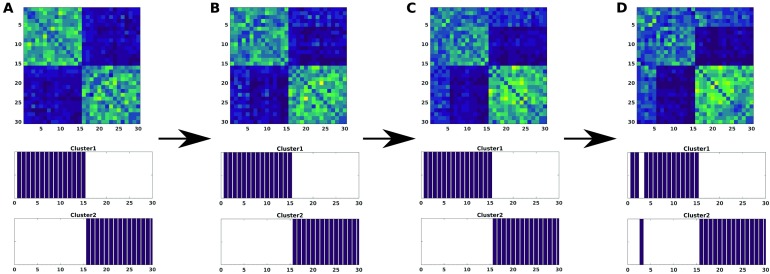
The modularity matrix among subjects of both groups and its cluster structure reconstruction provided by a Louvain community detection algorithm on this matrix is depicted when changing the number of components, i.e., the index *j* of *ϵ*^*ij*^ in Equation 6 affected by large amplitude noise (*A* = 0.5): *j* = (1) (A), *j* = (1…3) (B), *j* = (1…6) (C), *j* = (1…10) (D).

In a second scenario, variation of noise across subsets of components was studied. To amplify this effect, we restrict noise amplitudes to a fixed large value *A* = 0.5. Four subsets of components affected by noise were taken *j* = {(1), (1…3), (1…6), (1…10)}. In addition, in order to add more complexity, we considered the application of noise to five subjects of group 1 and 10 of group 2, respectively. As a consequence, this effect is stronger in group 2, since for the first 10 components the activity is lower and therefore more sensitive to noise. When applied to a different number of components, this leads to a decrease of intragroup distance of subjects affected (yellowish areas in the modularity matrices of Figure 3) and also a slight approximation between groups as a consequence of the synchronization among the noised components. Nevertheless, the consensus clustering is pretty robust when reconstructing both groups.

**Figure 4.**
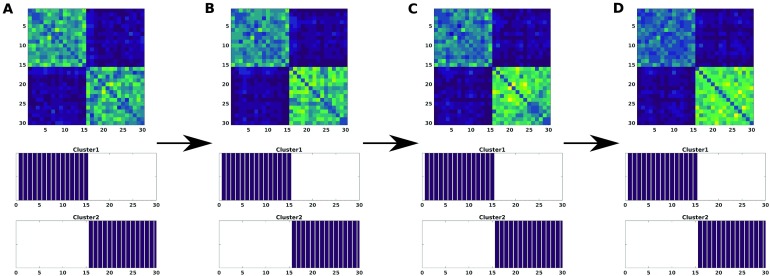
The modularity matrix among subjects of both groups and its cluster structure reconstruction provided by a Louvain community detection algorithm on this matrix is depicted when changing the subjects affected, i.e., the index *i* of *ϵ*^*ij*^ in Equation 6 affected by large amplitude noise (*A* = 0.5): *i* = (1) (A), *i* = (1…5) (B), *i* = (1…10) (C), *i* = (1…15) (D).

Finally, variation of number of subjects was taken into account (see Figure 4). To simplify this scenario, we considered that noise only affected group 2 and the first 10 components with a large amplitude *A* = 0.5. The subset of number of subjects are *i* = {(1), (1…5), (1…10), (1…15)}. The consensus turns out to be again robust when reconstructing both groups. For this case, the effect of changing the number of subjects of group 2 affected by noise makes this group more compact as more subjects are involved since noise makes them look more similar. This also makes these subjects of group 2 more similar with those of group 1 for the first 10 components, but not enough to mix them together to make them indistinguishable by the consensus method.

### LA5C dataset

**Figure 5.**
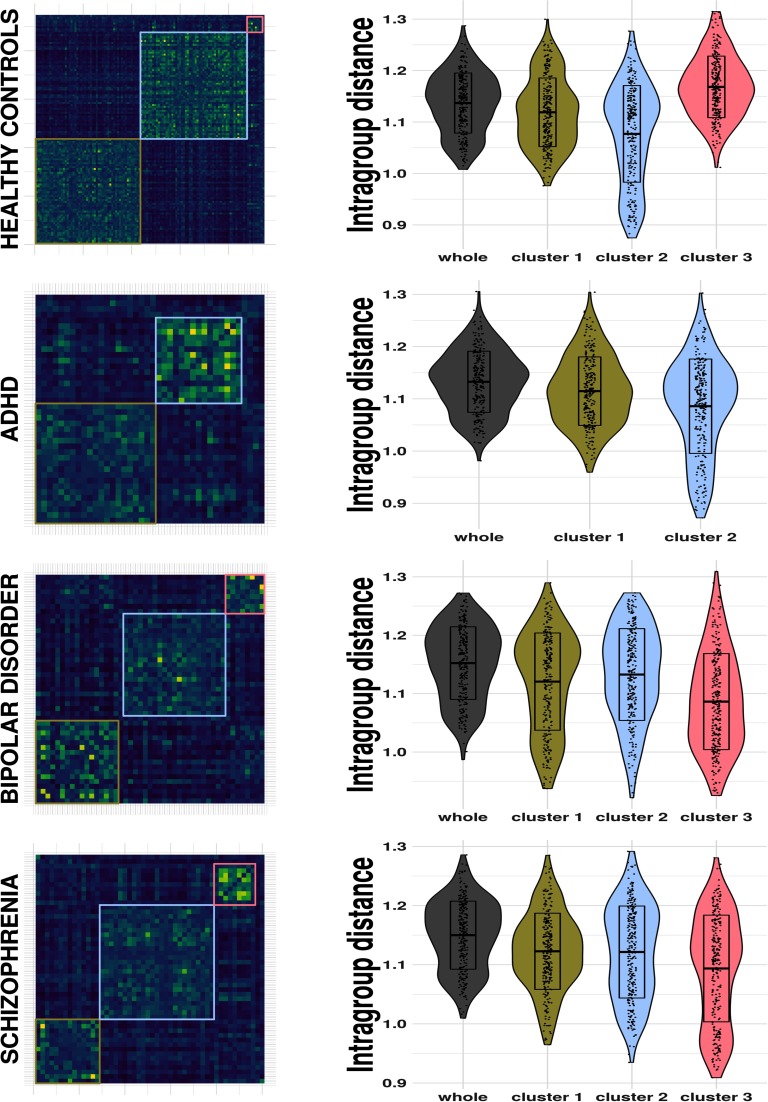
Modularity matrix and the reduction of the mean intragroup distance per node after applying the consensus clustering method for each group in the LA5C dataset.

The application of the consensus clustering method to the brain matrices in the four groups yields the modularity matrices depicted in Figure 5, which have been ordered according to the membership configuration found by the community Louvain routine. For the healthy group, this algorithm detects three communities of 55, 54, and 8, respectively. Such clusters are statistically differentiated by FWD (Kruskal-Wallis test, *p* < 0.0001) and sex (*χ*^2^ test, *p* = 0.0201). For the ADHD group, it finds two clusters of 21 and 15 subjects. For the BD group it detects three communities of 17, 21, and 8 subjects, and for the SCH group, three clusters of 14, 25, and 9, these SCH clusters being statistically different with respect to FWD (Kruskal-Wallis test, *p* = 0.0003). In all cases, communities with fewer subjects than five have been considered as outliers.

Likewise, the consensus clustering algorithm allows extraction of more homogeneous subgroups of subjects, as can be noticed from Figure 5, where the mean intragroup distance distribution produced by the collection of distance matrix for each pattern connectivity is exhibited. We stress that the intersubjects distance is highly and significantly smaller for the clusters with respect to the whole groups, as estimated pairwise by means of a Wilcoxon signed-rank test.

**Figure 6.**
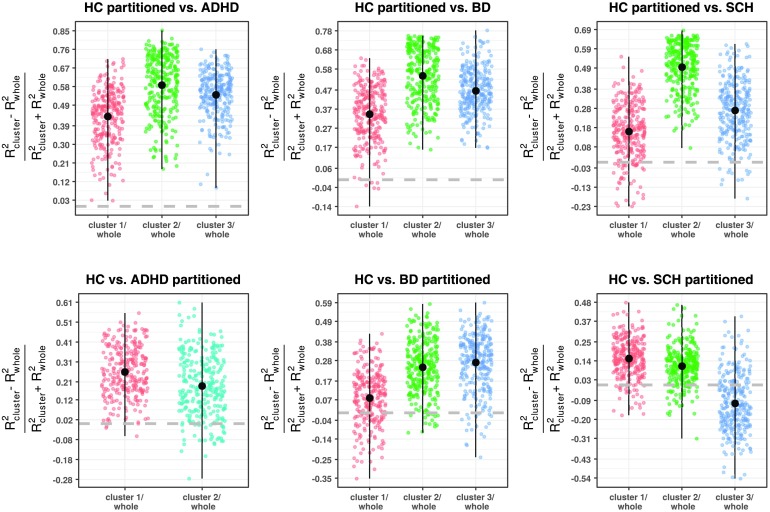
The change in the effect size measure *R*^2^ provided by MDMR across nodes, where subscript term “whole” involves both whole groups comparison and “cluster” one partitioned group by consensus clustering. An enhancement is developed above zero, here displayed by a gray thick line. Median value point reaching the maximum and minimum values are depicted here in black.

Furthermore, having more homogeneous clusters is translated into a variability decrease, leading in some cases to a gain in separability, when clusters are introduced in between-group comparison studies. As can be seen in Figure 6, the partition of individual groups by consensus clustering in general enhances the effect size (here evaluated through the pseudo-*R*^2^ measure in MDMR) per node associated with class difference. Furthermore, this also allows us to reject the null model even though the sample size decreases when a partitioned group is involved, providing a better control for type I errors compared with the unpartitioned case (see *p* values distribution Supporting Information Figure S2, Rasero, Diez, Cortes, Marinazzo, & Stramaglia, 2019). Concerning partition of the healthy group, such an enhancement is notorious, showing, for example, that a procedure selecting and recruiting subjects with similar characteristics of those subjects within the cluster 2 in our healthy control sample would optimize the node association with the pathology. In addition, for the ADHD group, which lacks of significant results when being involved in group comparisons as a whole, size effect amplification leads to development of a significant gain improvement when clustered using the consensus algorithm. In contrast, for both BD and SCH clusters such an improvement takes place specially in some of their clusters and as a consequence, the consensus algorithm can also help filter out “noisy” subjects, which have less in common with the pathology in study.

**Figure 7.**
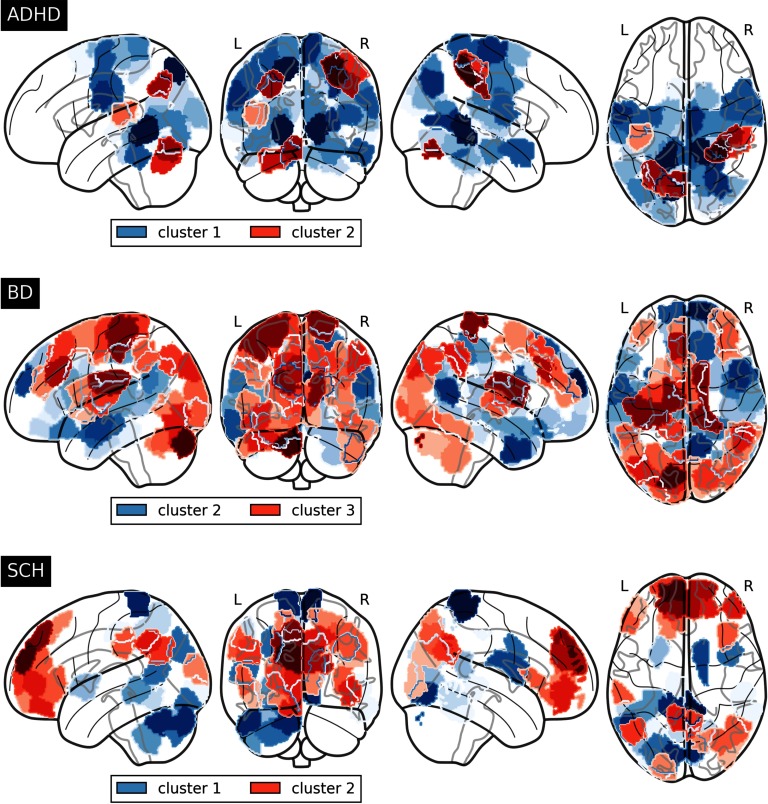
Unique nodes for each cluster within the disorder groups, i.e., significant nodes not presented in the whole group or the rest of cluster comparisons, which emerge when confronting with the whole healthy control group.

On the other hand, application of consensus clustering to brain connectivity matrices of pathologies can also help unmask substructures whose underlying properties are unique and that might be worth studying. For example, concerning the comparisons with healthy subjects of the ADHD group after having been partitioned, we obtain for cluster 1, 59 significant nodes that are present neither in cluster 2 nor when this group is taken as a whole. We will hereafter denote these nodes as *unique nodes* for cluster 1, meaning that only the comparison with cluster 1 leads one to point out their altered pattern with respect to pathological conditions. Likewise, seven nodes are unique for cluster 2 of the same disorder group. Regarding comparison with the BD group, there are 61 unique nodes that are recognized as significant for cluster 1 and 46 for cluster 2. Finally, 113 nodes arise as being significantly associated with the whole group of schizophrenia, whereas substructure inspection increases this number to 128 and 132 for clusters 1 and 2, exhibiting both 27 different unique nodes. Both cluster 1 of BD and cluster 3 of SCH keep subjects associated with both pathologies, and therefore no association is found whatsoever. Regions affected by unique nodes in each pathology are represented in Figure 7.

Higher significant regions for cluster 1 of ADHD lie in cortical regions from the lateral occipital cortex, superior division, the cingulate gyrus, posterior division and lingual gyrus, and involve functions from the visual and dorsal attention system. Cluster 2 has also incidence from parts of the lingual gyrus in addition to postcentral gyrus, the superior parietal lobule and the supramarginal gyrus, anterior division—all associated with the dorsal attention system—and a small portion of the cerebellum.

On the other hand, regarding the BD group, the default mode system fundamentally emerges in cluster 2 with cortical regions in the frontal pole, temporal pole, inferior temporal gyrus, anterior division and the precuneus. Cluster 3 includes the postcentral gyrus and the left thalamus, which take part in sensorimotor functions, and parts of the cerebellum and the occipital fusiform gyrus.

Finally, SCH community representation obtained through the consensus algorithm exhibits a well-differentiated structure, with cluster 1 focusing mainly on the posterior parts of the brain with the postcentral gyrus, precuneus and lingual gyrus, somatosensory, and visual systems; and cluster 2 involving basically the prefrontal cortex and some parts of the superior frontal gyrus (mainly associated to the default mode network).

### ABIDE dataset

**Figure 8.**
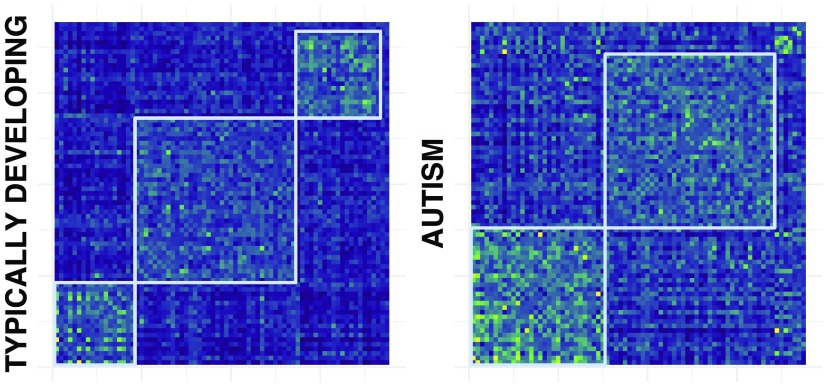
The modularity matrix *B* ordered after label structure provided by community detection is depicted for typically developing (left) and autism spectrum disorder (right) group in the ABIDE dataset considered.

**Figure 9.**
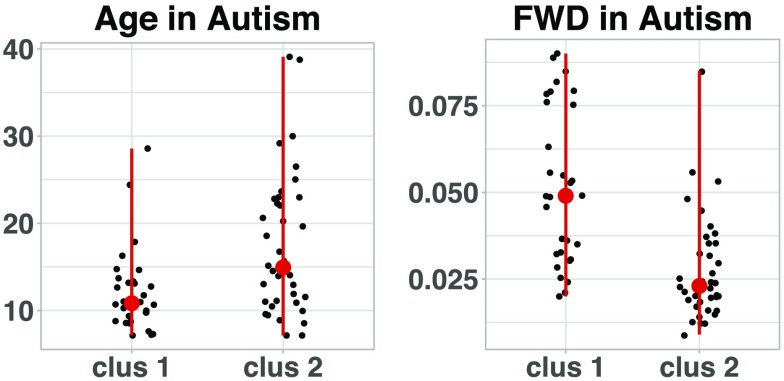
Age and FWD points distribution for the subjects within the clusters of ASD group found by the consensus clustering algorithm. In red, the dot displays the median, and the vertical lines reach the maximum and minimum dispersion of the points in each case.

Application of the consensus clustering algorithm and subsequent community detection algorithm to both groups leads to the ordered community structure provided by the modularity matrices in Figure 8. For the TD group, the modularity matrix *B* yields three modules of 18, 36, and 19 subjects, respectively. In contrast, division of the ASD group consists of two modules of 30 and 38 patients, respectively. Moreover, a Wilcoxon rank sum test performed on ASD community partition separates subjects statistically by age (*p* = 0.0028) and framewise displacement (*p* < 0.0001) (see Figure 9).

**Figure 10.**
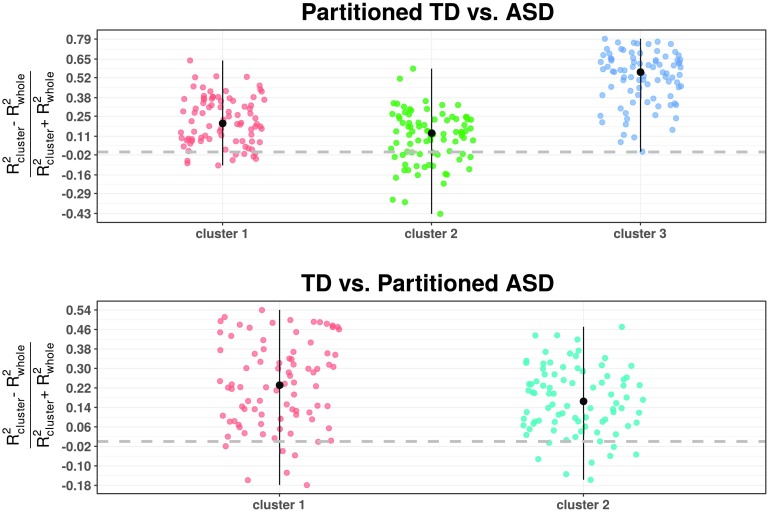
The change in the effect size measure *R*^2^ provided by MDMR across nodes, where subscript term “whole” involves both whole groups comparison and “cluster” one partitioned group. An enhancement is developed above zero, here displayed by a gray thick line. Median value point reaching the maximum and minimum values are depicted here in black.

**Figure 11.**
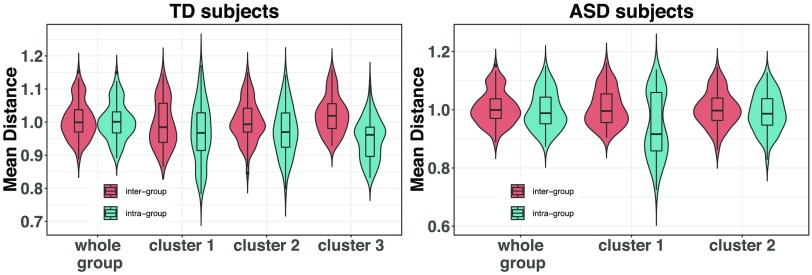
Mean intra- and intergroup distance distribution provided by the collection of distance matrices per each node. On the left, this is shown for the TD group, and on the right for the ASD subjects. The intergroup distance is always evaluated considering the other group as a whole.

On the other hand, we can see in Figure 10 that overall, the effect size of each node pattern connectivity for distinguishing between both group labels is larger when a partitioned case is involved. This is also translated into an increase of statistical significance (see Supporting Information Figure S3, Rasero et al., 2019). In particular, it is notable how cluster 3 of the TD population shows all the benefits of applying the consensus clustering method since it gathers the TD subjects with the highest connectome difference in comparison with the ASD subjects. Moreover, this pronounced case corresponds to the scenario where the decrease of intragroup distance (cluster is more homogeneous) also follows an increase of the intergroup distance (left panel of Figure 11). This effect is not reproduced in the rest of the cases, even though clusters become more compact. We are indeed clustering each group separately, thus maximum separation between classes does not need to be guaranteed. Likewise, the intragroup distance reduction also yields an effect size enhancement in both autistic subpopulations, more clearly for cluster 1 where this reduction is more pronounced. Consequently, the increase in effect size and the subsequent gain in statistical significance for some of the cases might allow to unmask regions not observed significantly different at first and therefore be further exploited by a more thorough exploratory analysis.

When considering both groups each as a whole, MDMR yields only two significant regions (region 57 and 83) as explained by group label variation between subjects, whereas this number increases to 13 and 70 for age and FWR, respectively, showing an important effect of the observed variance based on these last covariates. However, applying first consensus clustering to the TD group produces three clusters such that when comparing with the ASD group, one cluster yields 52 significant nodes, with 48 nodes uniquely assigned to it. Likewise, both clusters obtained by partitioning the ASD group lead to 16 and 9 significant nodes when compared with the group of typically developing subjects and, most importantly, 14 and 6 of them, respectively, only observable in each cluster. Therefore, not only we do gain separability by means of an increase of significant nodes, but also our approach allows us to unmask unique regions not visible whatsoever when using standard whole groups comparisons.

From a clinical point of view the emergence of unique nodes can evidence brain regions whose implications on the development of the pathology should be further examined. In our case, concentrating on the ASD case, cluster 1 has 14 regions not present neither in cluster 2 versus TD, nor in whole group comparison. These regions are prominently localized on the right brain hemisphere with the highest overall significance in the pars triangularis of the inferior frontal gyrus (*p*^fdr^ = 0.0004). Areas in the same hemisphere involving the frontal pole (*p*^fdr^ = 0.0018) and the precuneus (*p*^fdr^ = 0.0036) and postcentral (*p*^fdr^ = 0.0014) and paracentral regions (*p*^fdr^ = 0.0019) in the opposite hemisphere are also associated to greater separation. On the other hand, the six unique significant nodes of cluster 2 belong to areas in both hemispheres involving the banks of the superior temporal sulcus (*p*^fdr^ = 0.0021 in the left hemisphere and *p*^fdr^ = 0.0078 in the right one) and the superior temporal lobe (*p*^fdr^ = 0.0085 and *p*^fdr^ = 0.0075, respectively), and regions in the inferior temporal lobe (*p*^fdr^ = 0.0069) and the supramarginal (*p*^fdr^ = 0.0079) in the right hemisphere.

## DISCUSSION

New techniques aimed to increase group separability in neuroimaging studies are constantly being developed to gain deeper and better insight on brain function and malfunctioning. The use of connectomes as a biomarkers is also increasingly prominent. Our clustering method is naturally located in this latter field and helps unveil more compact subclasses beyond the group labels. In addition, the application of consensus clustering to different regions of the brain allows us to capture differences in the groups unseen when the whole connectivity matrix is used. To our best knowledge, such an approach has not been yet explored in neuroimaging. Additionally, the consensus matrix gathers information from different resolutions and nodes and encodes them into the consensus matrix. As a consequence, some possible arbitrariness such as choosing a priori the number of clusters to be obtained disappears. Moreover, clusters found by our method are fairly robust with respect to moderate changes in amplitude noise. Of note, clinical and control groups have been shown to share functional connectivity patterns (Easson, Fatima, & McIntosh, in press; Spronk et al., 2018). Here we do not go after a wholly data driven clustering of subjects irrespective of their main clinical label, but rather on refining these user-defined groups based on their FC patterns.

Preliminary analysis of groups of labeled subjects by consensus clustering leads to more compact partitions and a subsequent increase in separability of classes when performing group comparisons. This effect can be simply understood by looking at the comparison of two univariate distributions, where the separability of both groups can be expressed in terms of the mean difference (intergroup distance) divided by the variability of both groups (intragroup distance). As a consequence, a reduction of the intragroup distance can help increase separability. Nonetheless, it is important to note that application of our clustering method to an individual group only optimizes the intragroup distance, and therefore it might happen that for some partitions the intergroup distances also decrease, compensating the benefit of having more compact groups. Different strategies to preprocess the data and to address motion confounders modify to some extent the output of virtually any subsequent analysis. One of the most debated choices in this respect, and likely one of the most influential on the results, is global signal regression. In our original analyses we decided not to apply global signal regression because of the expected network-specific differential effects evidenced in Gotts et al. (2013). On the other hand, there are also reasons to use global signal regression (Murphy & Fox, 2017), and this choice was made in a recent paper proposing a similar subgroup classification strategy (Easson et al., in press). We repeated our analysis after regressing the global signal out, and found that the results were qualitatively similar (see Supporting Information Figure S4, Rasero et al., 2019 for the ABIDE dataset).

In addition to variability reduction that allows us to elucidate regions with distinctive connectivity patterns in group comparisons, each cluster found also provides unique information. In particular, we have shown this for two different datasets that include four different mental disorders: ADHD, BD, SCH, and ASD.

Most of the studies aimed at finding brain alterations in ADHD subjects have been focused on children, since the symptoms related to this disorder such as inattention and/or hyperactivity/impulsivity need to be treated at the beginning of their appearance. In our case, however, our cohort consists of adult subjects, among which we have found two different clusters carrying both different and unique information. The first cluster comprises a vast anterior brain area, in addition to the lateral occipital cortex and the lingual gyrus, mainly associated with visual attention. Similar results were reported using structural data (Ahrendts et al., 2011). The second one covers a small portion of the brain associated with the dorsal attention and the cerebellum, possibly capturing the cognitive functions of the latter in attention tasks (Berquin et al., 1998).

Our clustering method clearly evidences the default mode network as one of the relevant systems in one of the subgroups in bipolar disorder and schizophrenia. Such a fundamental network is likely very sensitive to most modulations and already reported to be affected in both mental disorders (V. Calhoun et al., 2012; Ongür et al., 2010). In our specific clusters, regions with a disrupted connectivity pattern concentrate in the frontal cortex (Deakin et al., 1989; Johnston-Wilson et al., 2000), with also some areas in the temporal lobe, which may account for differences between bipolar disorder and schizophrenia (Altshuler et al., 2000; V. D. Calhoun, Maciejewski, Pearlson, & Kiehl, 2008; Johnstone et al., 1989).

The present study evidenced areas involved in different functional systems, such as the cerebellum (DelBello, Strakowski, Zimmerman, Hawkins, & Sax, 1999; Forlim et al., 2017; McDonald et al., 2005), and the postcentral gyrus was connected to the appearance of delusion and hallucinations associated with a dysfunction of the sensory system (Song et al., 2015), and the bilateral lingual gyrus, whose hyperactivation has been connected to lack of empathy in schizophrenic subjects (Kühn & Gallinat, 2013).

On the other hand, two clear macroregions characterize our results on ASD subjects. The first one has a clear right asymmetry, with the participation of areas around the pars opercularis and pars triangularis in the inferior frontal gyrus, usually connected to lack of emotions (Dapretto et al., 2005; De Foss et al., 2004; Herbert et al., 2002). This domain also includes sensorimotor areas such as the postcentral and paracentral gyrus and precuneus and confirm the results observed in a larger ABIDE cohort of subjects when comparing ASD with typically developing controls (Lee, Kyeong, Kim, & Cheon, 2016). The second set of regions comprises some parts of the temporal lobe that, in addition to regions in the orbitofrontal cortex and the amygdala, define the “social brain.” In this case, autistic subjects develop abilities for verbally labeling complex visual stimuli and processing faces and eyes, to compensate for amygdala abnormality (Baron-Cohen et al., 1999).

It is worth stressing that the connectivity patterns among brain regions are influenced by respiration, movement, cardiac phase, and other physiological variables, and what we observe and can infer here (and in most fMRI studies) is just a higher level view in which the effects of all these actors are conflated.

## CONCLUSIONS

In this work we have proposed the use of the consensus clustering approach for exploratory analysis in order to cope with the heterogeneity of subjects. Extracting the natural classes present in data and subsequently performing the supervised analysis by MDMR on these allows us to identify variables whose adjacency pattern is altered in group comparisons, and which are not highlighted when considering the groups as a whole. As a result, the proposed approach leads to an increase in the effect sizes. We presented an application to public fMRI data, evidencing groups of subjects in which specific regions contributed to a higher separability between classes. In sum, we have shown an additional benefit of using the consensus clustering algorithm developed in Rasero et al. (2017). Such an algorithm is based on the idea of condensing information on structure at different levels, which in general works well when extracting the inherent data subclasses. Future studies could test alternative strategies when this is not the case.

## AUTHOR CONTRIBUTIONS

Javier Rasero: Conceptualization; Data curation; Formal analysis; Investigation; Methodology; Software; Validation; Visualization; Writing – original draft. Ibai Diez: Data curation. Jesus M Cortes: Conceptualization; Methodology; Supervision; Writing – original draft. Daniele Marinazzo: Conceptualization; Data curation; Formal analysis; Investigation; Methodology; Supervision; Visualization; Writing – original draft. Sebastiano Stramaglia: Conceptualization; Formal analysis; Investigation; Methodology; Supervision; Writing – original draft.

## FUNDING INFORMATION

Sebastiano Stramaglia, University of Bari “Aldo Moro” (http://doi.org/10.13039/501100005362), Award ID: “Progetti di Ricerca su base competitiva, f.di ex 60% - residuo 2015.” Javier Rasero, Department of Education (Basque Country Government), Award ID: Doctoral Research Staff Improvement Programme.

## TECHNICAL TERMS

Brain connectivity network (connectome): A network in which the nodes are brain regions and the links are anatomical connections (“anatomical/structural connectivity”) or statistical dependencies (“functional connectivity”).
Distance matrix: For each node, a distance matrix for the set of subjects is constructed as follows. For each pairs of subjects, the corresponding distance is $\sqrt{2(1-r)}$, where *r* is the Pearson correlation between the nodal connectivity patterns of the given node in the two subjects.
Consensus matrix: Given several partitions of a given set of nodes, for each pair of nodes the consensus matrix assumes, as the corresponding entry, the fraction of partitions in which the two nodes belong to the same subset.
Resting-state fMRI: Functional magnetic resonance imaging acquired while the subject is simply instructed to stay awake.
*K*-medoids algorithm: A classical partitioning technique of clustering that groups the data set of *n* objects into *k* clusters known a priori. Like the *k*-means, *k*-medoids algorithms attempts to minimize the distance between points labeled to be in a cluster and a point designated as the center of that cluster; in contrast to the *k*-means algorithm, *k*-medoids chooses data points as centers (medoids).
Modularity matrix: For each entry in the consensus matrix, the null ensemble of all possible configurations by label permutation at a given resolution is subtracted.
Pseudo-*R*^2^ measure: Measure that accounts for the proportion of sum of squared pairwise distances explained by the predictors and that can be therefore used to assess the effect size of association between the dependent and independent variables in the multivariate distance matrix regression.
