# Supplementary material for: Connectome sorting by consensus clustering increases separability in group neuroimaging studies
Source: Netw Neurosci. 2019 Feb 1;3(2):325–43. doi: 10.1162/netn_a_00074 (PMC6370473; doi:10.1162/netn_a_00074)
Supplement: Supplementary file 1 [file netn-03-325-s001.pdf]

Rasero, J., Diez, I., Cortes, J. M., Marinazzo, D., Stramaglia, S. (2019). Supporting information for "Connectome sorting by Consensus Clustering increases separability in group neuroimaging studies." *Network Neuroscience*, *3*(2), 325–343. https://doi.org/10.1162/netn_a_00074

- In Figure S1, the different mean inter-group distance distributions across nodes are depicted, showing that in some cases this distance can also increase in group comparison involving clusters. Therefore, this effects aids the intra-group reduction in doing the effect size enhancement more notorious.

- In Figure S2, we show the behaviour of the empirical cumulative distribution function along with the histogram distribution for the uncorrected p-values from each possible comparison between whole and cluster comparison within a control versus abnormal group setup. As we can see, the comparisons when no clustering has been performed begin to accumulate larger p-values in contrast to the case when some of the clusters are involved.

- In Figure S3, the uncorrected p-values histogram distribution from each possible comparison between whole and cluster comparison involved in each ABIDE group is depicted. As we can see, the comparisons when no clustering has been performed lead to have larger p-values, in contrast to the case when some of the clusters are involved.

- In Figure S4, the results from the application of our method prior to group-comparison analysis for the ABIDE dataset including now global signal regression in the preprocessing pipeline are depicted. As we can observe, the same conclusions as those without global signal regression are reached.

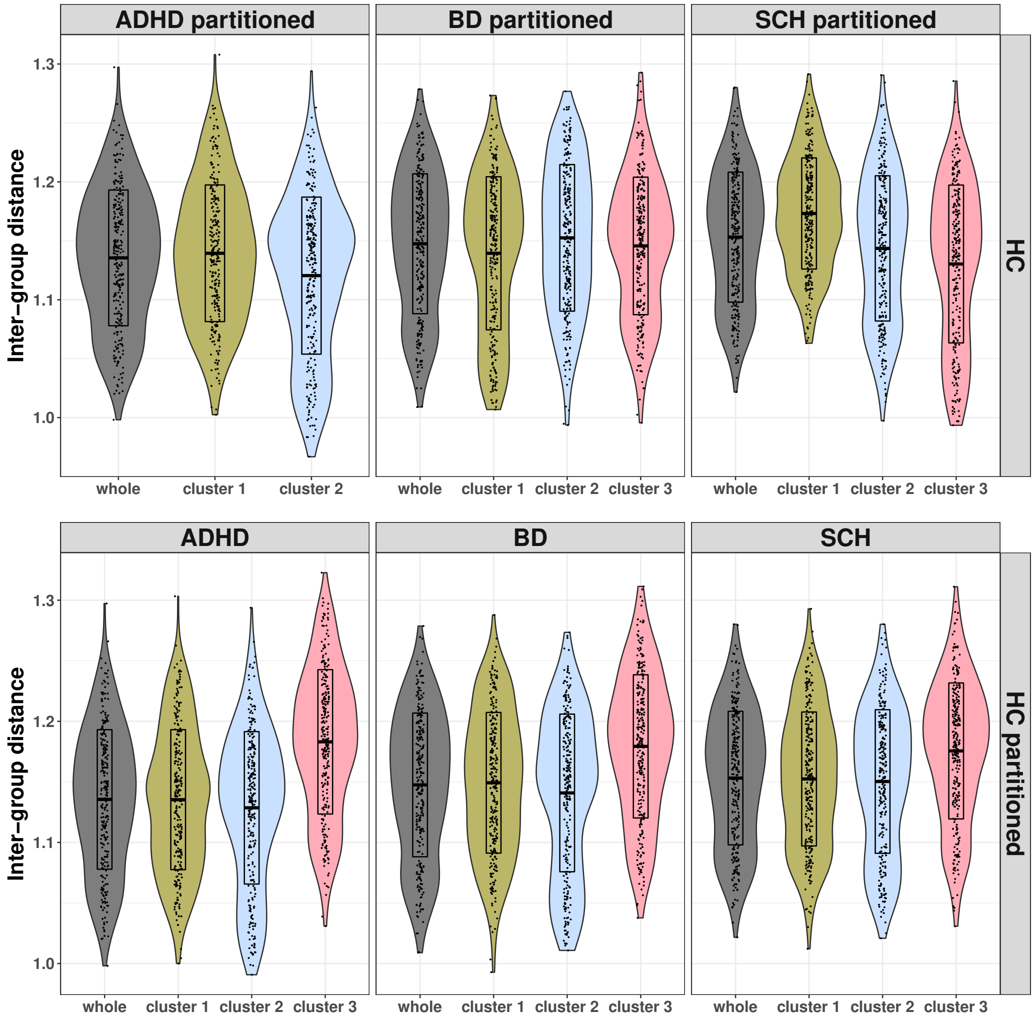

Figure S1: The mean inter-group distance across nodes distribution from all possible comparisons involving the whole groups and one partitioned group.

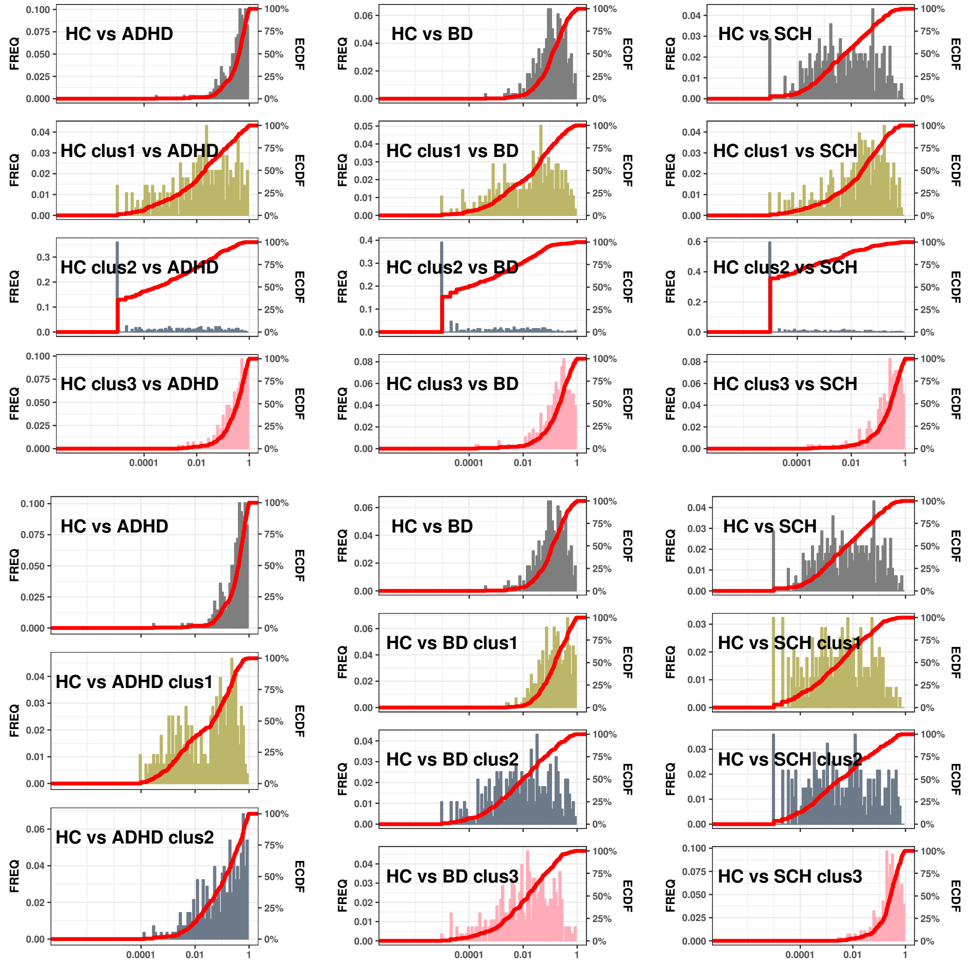

Figure S2: The empirical cumulative function along with the histogram distribution for the uncorrected p-values are displayed for all possible comparisons involving the whole groups and one partitioned group.

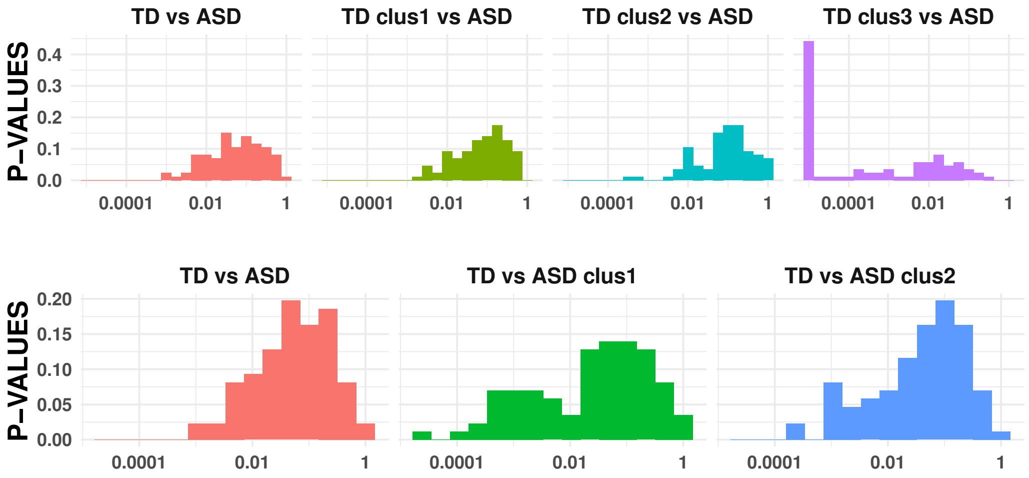

Figure S3: The uncorrected p-values distribution from comparing the whole groups (here denoted as TD ≡ Typically Developing and ASD ≡ Autism Spectrum Disorder) in the ABIDE dataset is depicted together with the same distribution when a partitioned group from the application of the consensus clustering algorithm is involved.

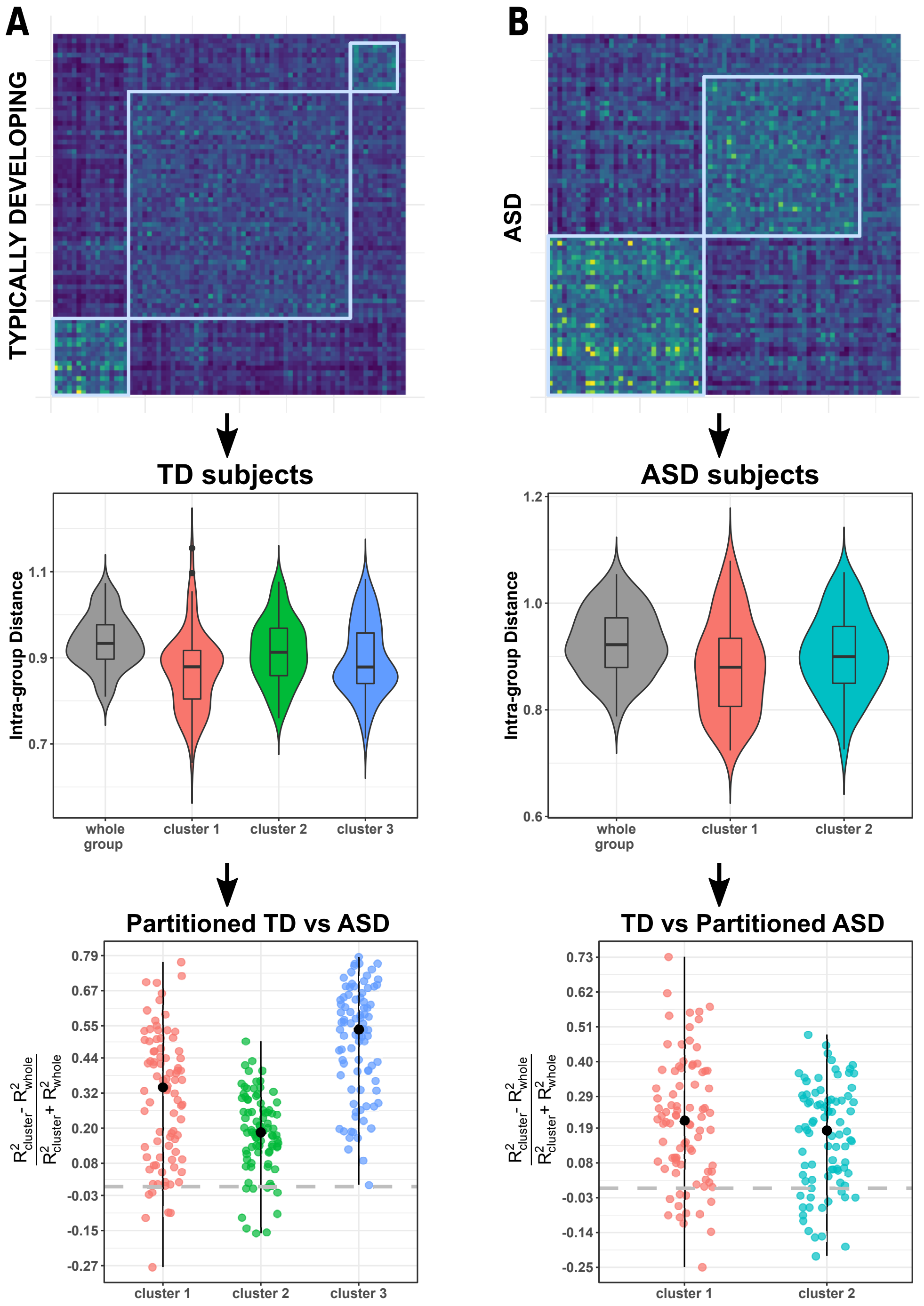

Figure S4: Results for the ABIDE dataset including the global signal regression step in the preprocessing pipeline. Panel A displays the modularity matrix, the intra-group distance and the effect size enhancement from partitioning the Typically Developing group. Panel B shows the same for the the partition of the Autism Spectrum Disorder group.
